# X-Linked TLR7 Deficiency Underlies Critical COVID-19 Pneumonia in a Male Patient with Ataxia-Telangiectasia

**DOI:** 10.1007/s10875-021-01151-y

**Published:** 2021-10-23

**Authors:** Hassan Abolhassani, Ahmad Vosughimotlagh, Takaki Asano, Nils Landegren, Bertrand Boisson, Samaneh Delavari, Paul Bastard, Maribel Aranda-Guillén, Yating Wang, Fanglei Zuo, Fabian Sardh, Harold Marcotte, Likun Du, Shen-Ying Zhang, Qian Zhang, Nima Rezaei, Olle Kämpe, Jean-Laurent Casanova, Lennart Hammarström, Qiang Pan-Hammarström

**Affiliations:** 1 Department of Biosciences and Nutrition, Karolinska Institutet, 14183 Huddinge, Sweden; 2 Research Center for Immunodeficiencies, Pediatrics Center of Excellence, Children’s Medical Center, Tehran University of Medical Sciences, Tehran, Iran; 3 Department of Pediatrics, North Khorasan University of Medical Sciences, Bojnurd, Iran; 4 St. Giles Laboratory of Human Genetics of Infectious Diseases, Rockefeller Branch, The Rockefeller University, New York, NY USA; 5 Department of Medical Biochemistry and Microbiology, Uppsala University, Uppsala, Sweden; 6 Centre for Molecular Medicine, Department of Medicine (Solna), Karolinska Institute, Stockholm, Sweden; 7 Laboratory of Human Genetics of Infectious Diseases, Necker Branch, INSERM U1163, Necker Hospital for Sick Children, Paris, France; 8 University of Paris, Imagine Institute, Paris, France; 9 Department of Laboratory Medicine, Karolinska Institute at Karolinska University Hospital Huddinge, Stockholm, Sweden; 10 Department of Endocrinology, Metabolism and Diabetes, Karolinska University Hospital, Stockholm, Sweden; 11 Howard Hughes Medical Institute, New York, NY USA

**Keywords:** COVID-19, critical COVID-19, inborn errors of immunity, primary immunodeficiency, antibody deficiency, ataxia-telangiectasia, ATM, TLR7

## Abstract

**Background:**

Coronavirus disease 2019 (COVID-19) exhibits a wide spectrum of clinical manifestations, ranging from asymptomatic to critical conditions. Understanding the mechanism underlying life-threatening COVID-19 is instrumental for disease prevention and treatment in individuals with a high risk.

**Objectives:**

We aimed to identify the genetic cause for critical COVID-19 pneumonia in a patient with a preexisting inborn error of immunity (IEI).

**Methods:**

Serum levels of specific antibodies against the virus and autoantibodies against type I interferons (IFNs) were measured. Whole exome sequencing was performed, and the impacts of candidate gene variants were investigated. We also evaluated 247 ataxia-telangiectasia (A-T) patients in the Iranian IEI registry.

**Results:**

We report a 7-year-old Iranian boy with a preexisting hyper IgM syndrome who developed critical COVID-19 pneumonia. IgM only specific COVID-19 immune response was detected but no autoantibodies against type I IFN were observed. A homozygous deleterious mutation in the *ATM* gene was identified, which together with his antibody deficiency, radiosensitivity, and neurological signs, established a diagnosis of A-T. Among the 247 A-T patients evaluated, 36 had SARS-CoV-2 infection, but all had mild symptoms or were asymptomatic except the index patient. A hemizygous deleterious mutation in the *TLR7* gene was subsequently identified in the patient.

**Conclusions:**

We report a unique IEI patient with combined ATM and TLR7 deficiencies. The two genetic defects underlie A-T and critical COVID-19 in this patient, respectively.

**Supplementary Information:**

The online version contains supplementary material available at 10.1007/s10875-021-01151-y.

## Introduction

The coronavirus disease 2019 (COVID-19) pandemic, which is caused by severe acute respiratory syndrome coronavirus 2 (SARS-CoV-2), has already affected more than 240 million people around the world [1]. This zoonotic enveloped virus is mainly transmitted by inhalation and infected people are usually asymptomatic, or have mild upper respiratory disease, and more rarely non-hypoxemic (intermediate) pneumonia. In some patients, however, it may lead to hypoxemic (severe) pneumonia and even acute respiratory distress syndrome (critical pneumonia) [2]. Globally, the case fatality ratio is around 2.0% (https://covid19.who.int/). The main epidemiological risk factor for the severe/critical forms of the disease is age, with a risk doubling every 5 years from childhood onward. Other risk factors, including male sex, higher body mass index, and other underlying cardiovascular, renal, and respiratory diseases, are much more modest [3].

The human genetic makeup and immunological status may also contribute to disease susceptibility or severity. Using genome-wide association studies (GWAS), multiple genetic loci that are significantly albeit moderately associated with SARS-CoV-2 infection or severe manifestations of COVID-19 have been identified, with odds ratios (OR) < 2.2 and typically < 1.5 [4]. Studies based on whole-genome/exome sequencing analyses have further suggested that rare inborn errors of immunity (IEI), including TLR3- and IRF7-dependent type I interferon (IFN) immunity, can cause life-threatening COVID-19 pneumonia [5]. Moreover, X-linked recessive TLR7 deficiency accounts for about 1% of cases in male patients younger than 60 years [6]. In contrast, auto-Abs neutralizing type I IFN accounts for about 15% of critical cases across ages, but 20% in patients > 80 years, and 20% of deaths across all ages [7, 8]. This is due to a sharp increase of auto-Abs neutralizing type I IFN after the age of 60 years, reaching 4% after 70 years and 7% between 80 and 85 years [8, 9].

IEIs other than those disrupting type I IFN have not been reported to underlie critical COVID-19. Among them, ataxia-telangiectasia (A-T) is a rare, pleiotropic, autosomal recessive IEI due to *ATM* gene mutations, presenting with cerebellar degeneration with ataxia, ocular and cutaneous telangiectasias, progressive childhood-onset immunodeficiency, cancer susceptibility, and radiation sensitivity [10]. A-T patients may exhibit a combined immunodeficiency with recurrent infections, neurological complications and a shortened lifespan [11, 12]. However, their potential susceptibility to SARS-CoV-2 infection is still unknown [12, 13]. Herein, we present the detailed clinical, immunological and genetic characterization of a unique IEI patient with critical COVID-19 pneumonia, who has pathogenic mutations in both X-linked *TLR7* and autosomal *ATM* (reported as P6 in a large COVID-19 cohort, with a *TLR7* mutation [6]).

## Methods

### Study Design

This study was conducted in accordance with a project of evaluation of critically ill IEI patient due to SARS-CoV-2 infection, prospectively enrolled in the Iranian national registry [12, 14, 15]. Critical cases were individuals who were admitted to the intensive care unit (ICU) due to respiratory failure, septic shock, and/or multiple organ dysfunction [1]. This study received approval from the Ethics Committee of the Tehran University of Medical Science. Moreover, written informed consent has been obtained from all patients, their parents, or legal guardians. The clinical diagnosis of the A-T was made according to the criteria of the European Society for Immunodeficiencies (ESID) [16]. The clinical characteristics of the index patient were compared with other registered A-T patients diagnosed based on ESID criteria and followed by scheduled visits every 1–3 months in the national IEI centers/hospitals according to the consensus guideline [17]. Genetic diagnoses were performed on A-T patients who agreed with the process of whole-exome sequencing (WES). Screening of COVID-19 were conducted on all registered IEI patients as previously described during the pandemic using reverse transcriptase-polymerase chain reactions (RT-PCR) and high-resolution computed tomography (HRCT) as well as other basic laboratory tests [12].

### Genetic Analysis and Diagnoses

Genomic DNA was extracted from whole blood from the index patient and WES was performed to detect single nucleotide variants, insertion/deletions and large deletions using a pipeline described previously [18, 19]. Candidate variants were evaluated by the Combined Annotation Dependent Depletion (CADD) algorithm and an individual gene cutoff given by using the Mutation Significance Cutoff (MSC) was considered for impact predictions [20]. The pathogenicity of all disease attributable gene variants was re-evaluated using the updated guideline for interpretation of molecular sequencing by the American College of Medical Genetics and Genomics (ACMG) criteria [21, 22], considering the allele frequency in the population database, *in silico *prediction, immunological data and clinical phenotyping.

### Detection of Autoantibodies Against IFNs

Serum samples were screened for autoantibodies against multiple IFNs using a bead array. Recombinant human proteins (Origene CAT# TP721103: IFNA1, TP321091: IFNA2, PBL11101-2: IFN-A2a, TP323649: IFNA4, TP310825: IFNA5, TP760329: IFNA6, TP311169: IFNA8, TP314055: IFNA10, TP320824: IFNA17, TP310115: IFNA21, TP723168: IFNW1, DA3547: IL6, LC400789: IFNB1, and TP721239: IFNG) were coupled to magnetic beads (MagPlex®, Luminex Corp.) containing different fluorescence markers. The AMG Activation Kit for Multiplex Microspheres (CAT#A-LMPAKMM-40) was used for coupling 500,000 beads with 1 μg of each protein according to the manufacturer’s protocol. Samples were heat-inactivated at 56 °C for 30 min, diluted 1:250, and randomly distributed in 96-well microtiter plates. The diluted samples and coupled beads were incubated together on a shaker in 384-well assay plates (Greiner Bio-One) at room temperature for 2 h. Autoantibody binding was detected using a R-phycoerythrin-labeled goat anti-Human IgG secondary antibody (eBioScience CAT#12–4998-82), and the FlexMap 3D instrument (Luminex Corp).

The blocking activity of anti-IFN-α2 and anti-IFN-ω auto-Abs was determined with a reporter luciferase activity. Briefly, HEK293T cells were transfected with a plasmid containing the Firefly luciferase gene under the control of the human ISRE promoter in the pGL4.45 backbone, and a plasmid constitutively expressing Renilla luciferase for normalization (pRL-SV40). Cells were transfected in the presence of the X-tremeGene9 transfection reagent (Sigma-Aldrich, ref. number 6365779001) for 24 h. Cells in Dulbecco’s modified Eagle medium (DMEM, Thermo Fisher Scientific) supplemented with 2% fetal calf serum (FCS) and 10% healthy control or patient serum/plasma (after inactivation at 56 °C, for 20 min) were either left unstimulated or were stimulated with IFN-α2 (Milteny Biotec, ref. number 130–108-984), IFN-ω (Merck, ref. number SRP3061), at 10 ng/mL or 100 pg/mL, or IFN-β (Milteny Biotech, ref. number: 130–107-888) at 10 ng/mL, for 16 h at 37 °C. Each sample was tested once for each cytokine and dose. Finally, cells were lysed for 20 min at room temperature and luciferase levels were measured with the Dual-Luciferase® Reporter 1000 assay system (Promega, ref. number E1980), according to the manufacturer’s protocol. Luminescence intensity was measured with a VICTOR-X Multilabel Plate Reader (PerkinElmer Life Sciences, USA). Firefly luciferase activity values were normalized against Renilla luciferase activity values. These values were then normalized against the median induction level for non-neutralizing samples, and expressed as a percentage. Samples were considered neutralizing if luciferase induction, normalized against Renilla luciferase activity, was 11% of the median values for controls tested the same day.

### Expression Vectors, Transfection Experiments, Western Blotting, and Luciferase Reporter Assay

*The TLR7* variant of the patient in our analysis was generated by site-directed mutagenesis. The WT or variant allele (L372M) was re-introduced into a Myc-DDK-pCMV6 vector (Origene). HEK293T cells, which have no endogenous TLR7 expression, were transfected with the Myc-DDK-pCMV6 vector, empty or containing the WT or a variant allele (L372M), in the presence of X-tremeGENE™ 9 DNA Transfection Reagent (Sigma-Aldrich), according to the manufacturer’s instructions.

For whole-cell extracts, the cells were lysed by incubation in the following buffer (50 mM Tris–HCl, pH 8.0, 150 mM NaCl, 1% NP40), supplemented with a mixture of protease inhibitors (Sigma-Aldrich), for 30 min at 4 °C. The lysates were then centrifuged at 21,000 × *g* for 20 min at 4 °C. The supernatants were processed directly for western blotting. Western blotting was performed on 10 µg of total extract from transfected HEK293T cells, with monoclonal antibodies specific for the leucine-rich repeats to the N-terminus within the human TLR7 protein (Cell Signaling Technology; clone, D7).

HEK293T cells, were transfected with the pCMV6 vector bearing wild-type or variant *TLR7* (50 ng), the reporter construct pGL4.32 (100 ng), and an expression vector for *Renilla* luciferase (10 ng), with the X-tremeGENE™ 9 DNA Transfection Reagent kit (Sigma-Aldrich). The pGL4.32 [luc2P/NF-κB-RE/Hygo] (Promega) reporter vector contains five copies of the NF-κB-responsive element (NF-κB-RE) linked to the luciferase reporter gene *luc2P*. After 24 h, the transfected cells were left unstimulated or were stimulated with 1 μg/mL R848 (Resquimod) for activation via TLR7/8 (Invivogen), or 5 μg/mL CL264 (Invivogen) for human TLR7-specific agonists, for 24 h. Relative luciferase activity was then determined by normalizing the values against the firefly: *Renilla* luciferase signal ratio.

## Results

**Fig. 1:**
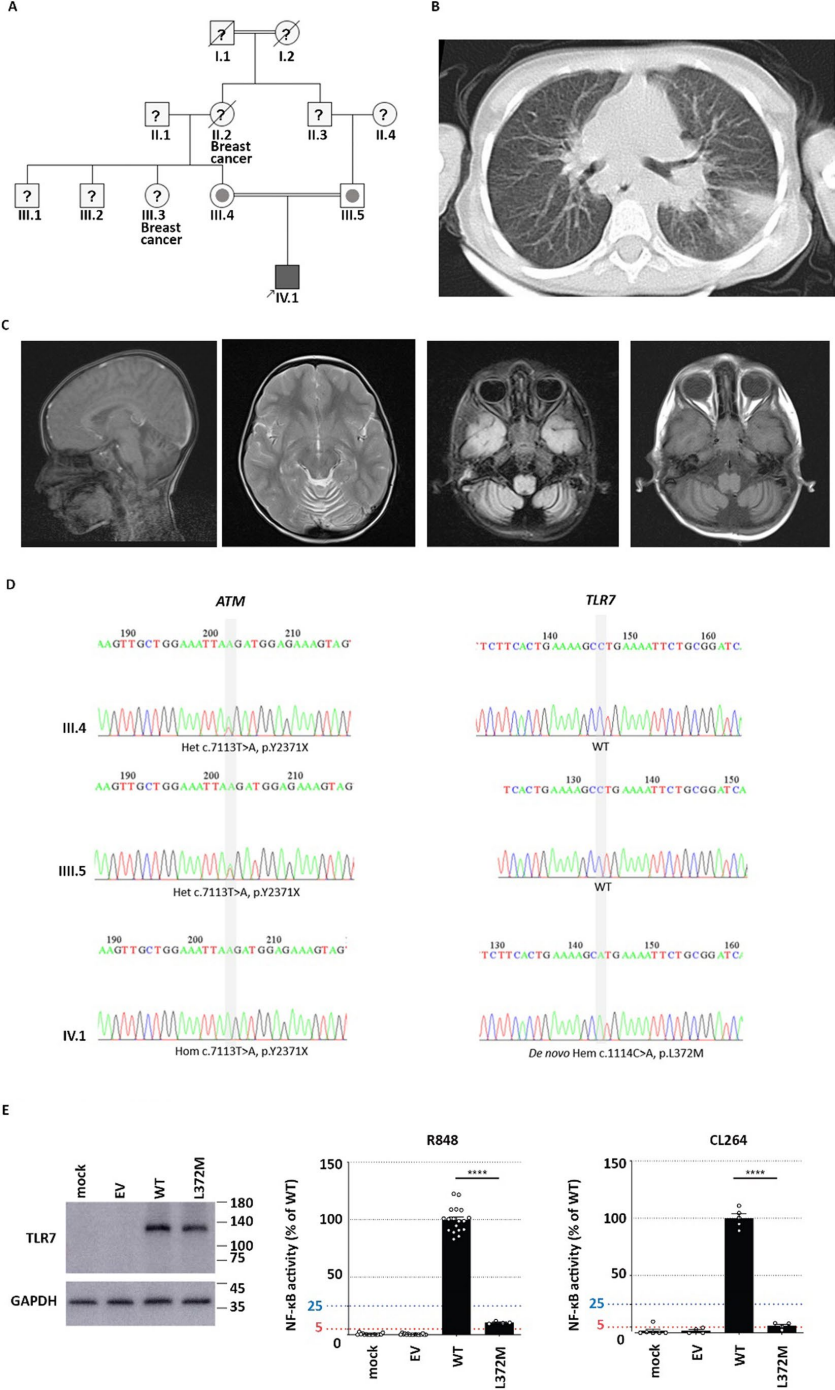
Clinical and genetic evaluation of a patient with combined ATM and TLR7 deficiencies associated with critical COVID-19 disease. Panel **a** shows the pedigree of the index patient; panel **b** shows computed tomography (CT) scan of the lung with opacification and dense consolidation on air bronchograms; panel **c** shows cerebral abnormalities in magnetic resonance imaging (MRI); panel **d** shows confirmatory Sanger sequencing in the proband and his parents; Panel **e** depicts the protein expression and functional activity of the TLR7 variant in response to resquimod (R848) and imidazoquinoline CL264

**Fig. 2:**
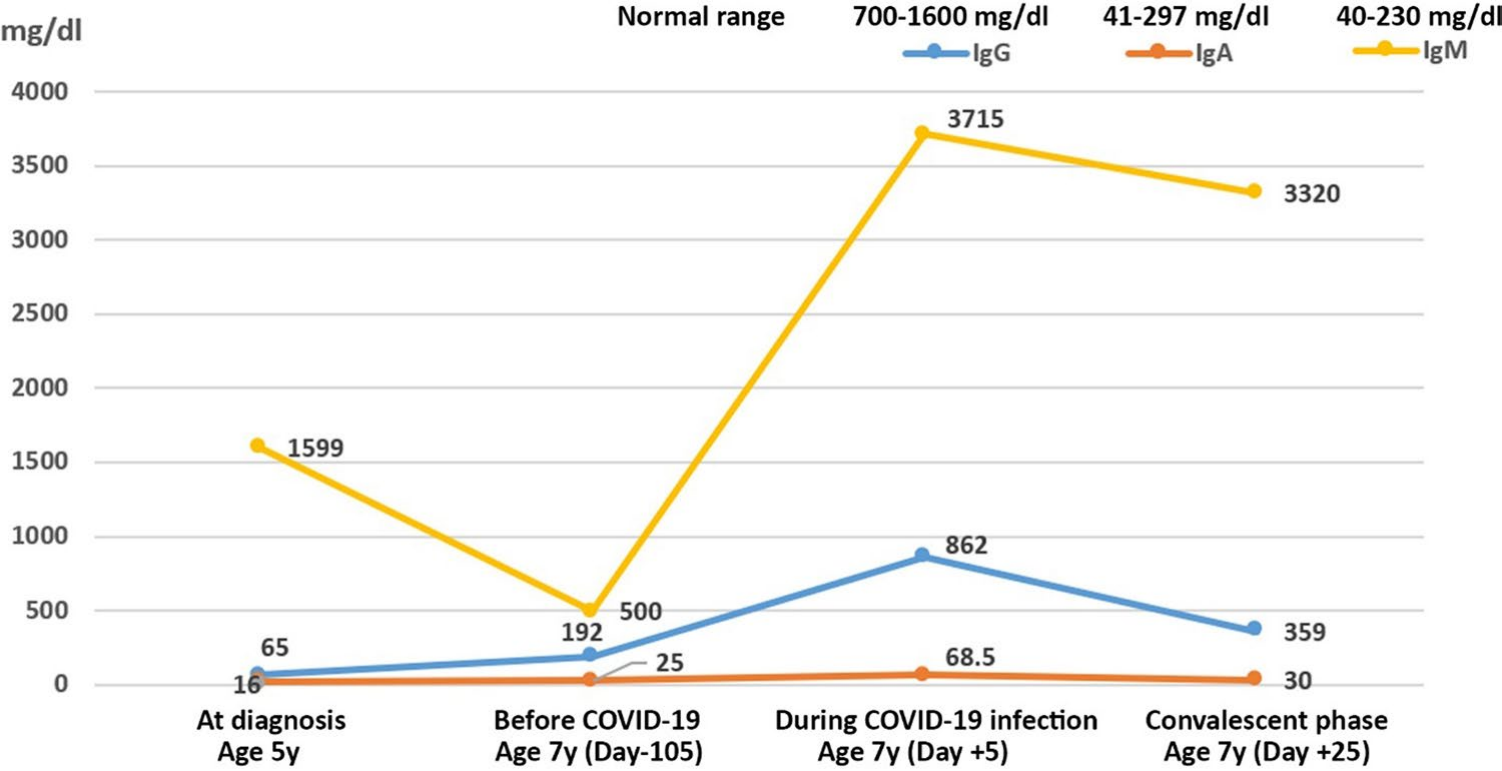
Transient normalization of IgG and IgA levels in the index patient during the acute phase of SARS-CoV-2 infection

The index patient is a 7-year-old boy born to first-cousin consanguineous healthy parents. There was no history of specific immune-related diseases in his family, but two breast cancer-affected individuals were reported (Fig. 1a). At the age of one year, he began to suffer from recurrent fever, upper respiratory tract infections and otitis media. Shortly afterwards, he developed severe pneumonia, which improved after broad-spectrum antibiotic treatment. At the age of 4, a failure to thrive (FTT) was noticed in which both his stature- and weight-for-age percentiles were below 3%. Later, he developed osteomyelitis of the hip and was admitted to the hospital. In the general examination of his FTT, splenomegaly, anemia, and thrombocytopenia were noted. He underwent a hip capsule biopsy, which revealed granulomatous tissue. The patient also underwent an endoscopy to check for celiac disease, which was ruled out, but a mild inflammation was reported in his duodenum and an autoimmune enteropathy was suspected. Immunological tests at this admission (age 5 years) showed that serum IgA and IgG levels were low, whereas IgM was high (Fig. 2), suggesting a diagnosis of a hyper immunoglobulin M (HIgM) syndrome, and intravenous immunoglobulin (IVIg) therapy was initiated.

Two years later, at age 7 years, he was re-examined due to another episode of upper respiratory infection and a slightly stiff, broad‐based gait. The result confirmed his previous HIgM diagnosis with low through-levels of IgG despite treatment with IVIg. A few months later, in September 2020, he went to the hospital emergency department with fever, cough, and symptoms of pneumonia. Complete blood count and erythrocyte sedimentation rate were carried out at the time of his arrival (Table 1). Further workup was performed, and he was diagnosed with COVID-19 pneumonia by RT-PCR test, and presence of consolidation in his chest computed tomography (Fig. 1b). Treatment with ceftriaxone, vancomycin, azithromycin, and chloroquine was initiated. Shortly afterwards, his serum immunoglobulins levels were analyzed. Although he had not received IVIg for the past month, his serum IgG and IgA levels were transiently increased during the early phase of SARS-CoV-2 infection (Fig. 2). Despite early admission to the infectious disease ward and initiation of IVIg, the condition of the patient deteriorated with severe seizures, hypertension, dilatation of the aorta, and renal failure at day 10 after infection. He was relocated to the pediatric intensive care unit (PICU) and after two days he was intubated and put on mechanical ventilation. He was discharged with improved symptoms after 25 days of treatment with parenteral nutrition, high-dose aspirin, corticosteroids (methylprednisolone), convalescent plasma infusion and anticoagulant (subcutaneous enoxaparin). In the follow-up, the patient's clinical presentation had returned to pre-infection status.

**Table 1:** Immunological profile of the index patient at day 8 after initial symptoms of COVID-19

Test	Result	Normal range for age
White blood cells (10^^^3/µL)	5.8	4–11
Platelets (10^^^3/µL)	74↓	150–450
Neutrophil (%)	70↑	20–45
Lymphocyte (%)	22↓	46–76
CD3^+^ T cells (%)	80↑	30–78
CD4^+^ helper T cells (%)	35	22–58
CD4^+^ CD45RA^+^ CCR7^+^ naïve helper T cells (%)	3.9↓	11–53
CD4^+^ CD45RA^−^ CCR7^+^ central memory helper T cells (%)	15	10–32
CD4^+^ CD45RA^−^ CCR7^−^ effector memory helper T cells (%)	65↑	0–40
CD4^+^ CD45RA^+^ CCR7^−^ TEMRA helper T cells (%)	6.1	5–15
CD4^+^ CD25^+^ FOXP3^+^ CD127^−/low^ regulatory T cells (%)	5.5	5–10
CD8^+^ cytotoxic T cells (%)	40↑	10–37
CD8^+^ CD45RA^+^ CCR7^+^ naïve cytotoxic T cells (%)	26	25–70
CD8^+^ CD45RA^−^ CCR7^+^ central memory cytotoxic T cells (%)	11↑	2–7
CD8^+^, CD45RA^−^ CCR7^−^ effector memory cytotoxic T cells (%)	43	15–60
CD8^+^, CD45RA^+^ CCR7^−^ T_EMRA_ cytotoxic T cells (%)	51↑	5–45
CD19^+^ B cells (%)	3.0*↓	6–14
Red blood cells (10^6/µL)	3.27↓	4.2–6.3
Hemoglobin (g/dl)	7.9↓	12–16
Hematocrit (%)	22.6↓	30–45
Mean corpuscular volume (fl)	69.1↓	80–100
Mean corpuscular hemoglobin (pg)	24.2↓	27–32
Mean corpuscular hemoglobin concentration (g/dl)	35.0	33–38
ESR 1 h (mm/h)	140↑	< 50
HIV DNA PCR	Negative	Negative
HIV antibody	0.3	< 1
50% hemolytic complement (CH50, U/ml)	8.8↓	50–150
Nitro blue tetrazolium test (%)	98	90–100
Alpha fetoprotein (ng/ml)	67↑	> 20

^*^B cell subset was not reliable in this patient due to low absolute count of B cells

Due to the severity of his COVID-19 disease and a previous history of antibody deficiency, a blood sample was collected for further immunological and genetic evaluations on the second day of PICU admission (day 12 after the first presentation). Levels of specific antibodies against the receptor-binding domain (RBD) of the spike (S) protein and the S protein of SARS-CoV-2 were measured using a previously described in-house ELISA method [23], and an isolated production of specific IgM antibodies was noted in the patient (Table S1). We next screened our index patient for serum autoantibodies against multiple interferon species using a bead array and all investigated autoantibodies against type I IFN species were negative (Table S2). Furthermore, using a luciferase reporter assay, we confirmed that patient serum lacks the neutralizing activity against IFN-α2 or IFN-ω. WES was subsequently performed and analyzed using a method described previously [18]. We identified a novel homozygous stop-gain mutation of the *ATM* gene (c.7113 T > A, p.Tyr2371Ter, Fig. 1d and Figure S1), which was predicted to result in a loss-of-function, truncated protein that lacks the entire kinase domain. Of note, A-T patients with HIgM may present with autoimmunity (due to IgM auto-antibodies levels) and lymphoproliferation (due to overactivation of B cells with a blockage at the germinal center stage) before a neurological presentation. These symptoms may appear in the absence of other systemic and typical symptoms, resulting in a delayed diagnosis and treatment. Furthermore, magnetic resonance imaging revealed signs of neurodegeneration in the patient, with moderate atrophy of cerebellar hemispheres, fusiform gyrus, enlarged 4th ventricle diameter and subarachnoid space fluid (Fig. 1c). Moreover, an elevated serum level of alpha-fetoprotein and increased radiosensitivity of patient cells further supported the diagnosis of A-T (Table 1 and Table S3).

Currently, 247 A-T patients (25 with a HIgM phenotype, 10.1%) have been registered in the Iranian IEI national registry [14]. Up to now, 36 patients (14.5%, including 5 unrelated cases with a HIgM phenotype, Table S4) have been confirmed to have COVID-19 and among these, 27 were asymptomatic, 8 showed mild symptoms, and only one, the index patient reported here, had a critical clinical presentation with ICU admission (*n* = 1, 2.7%; P1 in Table S4). We therefore further investigated the WES data for additional genetic defects that might be related to the severe COVID-19 presentation in this patient (Table S5) and identified a novel hemizygous mutation in the *TLR7* gene (c.1114C > A, p.Leu372Met, Figure S1). Sanger sequencing confirmed both mutations and that the *ATM* mutation was inherited from heterozygous parents whereas the *TLR7* variant was a de novo mutation (Fig. 1d). Of note, none of the sequenced A-T patients with or without COVID-19 (*n* = 29, including 4 of remaining HIgM patients with asymptomatic/mild presentation) carried variants in the *TLR7* gene. Western blot of extracts from HEK293T cells transfected with pCMV6 vector with the Leu372Met-TLR7 variant (Fig. 1e) showed that the TLR7 mutant was expressed at a normal level. However, NF-κB luciferase activity of these cell lines upon stimulation with Resiquimod (R848, an agonist of both TLR7 and TLR8) and imidazoquinoline CL264 (a TLR7-specific ligand) revealed only about 5–10% of activity of the L372M-TLR7 variant compared to wildtype, indicating that this variant was severely hypomorphic (Fig. 1e).

## Discussion

This is a critical COVID-19 case, with a pre-existing IEI condition. It is unknown if the antibody and T cell defects associated with the ATM deficiency may contribute to severe COVID-19. Viral infections have been reported in 25–30% of A-T patients mainly in the respiratory tract due to Rhinovirus and it is usually asymptomatic [13, 24]. Uncomplicated Varicella-zoster virus (VZV), Herpes simplex virus (HSV) and Molluscum contagiosum have also been reported in a minority of patients [25]. Although severe acute infections are uncommon [26], viral susceptibility has been reported in A-T patients with a HIgM phenotype [13]. However, as our other SARS-CoV-2 infected A-T patients, including those with a HIgM phenotype, did not have severe COVID-19 (Table S4), the *ATM* mutation identified here is probably underlying the primary antibody deficiency (HIgM) in this patient, but not the critical COVID-19. This notion is supported by previous observations among other SARS-CoV-2 infected IEI patients worldwide where almost no AT patient has been reported despite its high frequency compared to other IEI entities [27, 28]. In contrast, the *TLR7* mutation, which impairs the function of the protein (Fig. 1e), is most likely responsible for the severe COVID-19. Indeed, very rare variants in *TLR7* have been identified in patients with severe or critical COVID-19 patients [29, 30]. The most recent X chromosome-wide genetic study on a large cohort of patients with critical COVID-19 pneumonia (*n* = 1202) further showed deleterious *TLR7* mutations can be identified in about 1.8% of male patients younger than 60 years and a high penetrance of severe or critical COVID-19 in individuals with TLR7 deficiency [6]. Furthermore, the ability to produce type I IFN in response to SARS-CoV-2 was impaired in plasmacytoid dendritic cells from these TLR7 deficient patients, which mechanistically underlies the critical form of the disease [6]. Thus, as human TLR7 is essential for immunity against SARS-CoV-2 and TLR7 deficiency underlies life-threatening COVID-19 pneumonia, while A-T patients without TLR7 deficiency control SARS-CoV-2 well, our patient with both autosomal recessive A-T and X-linked recessive TLR7 deficiency had critical COVID-19 because of TLR7 deficiency. We nevertheless cannot exclude the possibility that the ATM deficiency modified the course of COVID-19 in our TLR7-deficient patient. Several studies have shown that DNA damage due to ATM deficiency primes the type I IFN system via the cytosolic DNA sensor STING [31, 32], which might provide robust antimicrobial responses and protect A-T patients from severe COVID-19. However, its combination with TLR7 deficiency might reverse such a phenomenon and lead to severe SARS-CoV-2 infection. Moreover SARS-CoV-2 infection induces sustained humoral immune responses which might be abrogated in A-T patients. Indeed, only an isolated IgM specific antibody response was detected in our patient. The potential synergistic impact of defective type I IFN and the humoral immune response should be considered in future studies.

Some patients have previously been reported with an inheritance of two monogenic IEI (*TNFRSF13B*-*TCF3* [33], *LRBA-NEIL3* [34], and *IFNAR1*-*IFNGR2* [35]). Of note, *IL12RB1* deficiency has been reported in an A-T patient with atypical features such as extraintestinal, nontyphoidal salmonellosis. These manifestations have never been identified in other A-T cohorts and the diagnosis of combined IL12RB1 and ATM deficiencies has certainly changed the management and treatment of the patient [36]. Our combined TLR7-ATM deficient case reported here further illustrates those two genetic defects can underlie two phenotypes. This observation has important clinical implications. Searching for a second, unknown pathogenic mutation should be performed, if a phenotype does not obviously match with the one specific IEI known in a patient.

## Supplementary Information

Below is the link to the electronic supplementary material.

Supplementary file1 (DOCX 491 KB)

## Data Availability

The raw data supporting the conclusions of this article will be made available by the authors, without undue reservation, to any qualified researcher.
