# Supplementary material for: X-Linked TLR7 Deficiency Underlies Critical COVID-19 Pneumonia in a Male Patient with Ataxia-Telangiectasia
Source: J Clin Immunol. 2021 Oct 23;42(1):1–9. doi: 10.1007/s10875-021-01151-y (PMC8536475; doi:10.1007/s10875-021-01151-y)
Supplement: Supplementary file 1 — Supplementary file1 (DOCX 491 KB) [file 10875_2021_1151_MOESM1_ESM.docx]

**Supplementary Data**

**X-linked TLR7 deficiency underlies critical COVID-19 pneumonia**

**in a male patient with ataxia-telangiectasia**

Abolhassani et al.

| **Table S1**- Anti-SARS-CoV-2 specific antibody response in the index patient. | | | | | | | | | | | | | |
| --- | --- | --- | --- | --- | --- | --- | --- | --- | --- | --- | --- | --- | --- |
| **S1-S2** | | | | | | **RBD** | | | | | | | |
| **IgM AU/ml** | | **IgA AU/ml** | | **IgG AU/ml** | | **IgM AU/ml** | | **IgA AU/ml** | | | **IgG AU/ml** | | |
| Patient | Cut off* | Patient | Cut off* | Patient | Cut off* | Patient | Cut off* | | Patient | Cut off* | | Patient | Cut off* |
| **38.3** | 2.5 | 0.00 | 0.5 | 0.00 | 0.03 | **14.8** | 8.4 | | 0.00 | 0.08 | | 3.8 | 14.8 |
| ** Cut off based on serum samples from pracademic controls (historic controls) and confirmed cased of COVID-19 ^1^.* | | | | | | | | | | | | | |

| **Table S2**- Serum sample of the index patient was screened for autoantibodies against multiple interferons as explained in the methods. | | |
| --- | --- | --- |
| **Parameters** | **ATM-TLR7 deficient patients (MFI)** | **Mean normal population**  **(MFI)** |
| IFNA21 | 38 | 126.9 |
| IFNA5 | 28 | 69.6 |
| IFNA4 | 37 | 102.8 |
| IFNW1 | 31 | 46.4 |
| IFNA6 | 88 | 439.2 |
| IFNA10 | 34 | 94.7 |
| IFNA8 | 126 | 349.1 |
| IFNA4*1 | 43 | 108.9 |
| IFNA17 | 34 | 66.7 |
| IFN A Alpha | 39 | 83.4 |
| IFNA2 | 39 | 160.9 |
| IFNA1 | 37 | 86.3 |
| IFNG | 31 | 57.4 |
| IFNB1 | 28 | 36.7 |
| GAD2 | 37 | 62.7 |
| IL22 | 30 | 77.3 |
| IL17F | 54 | 320.1 |
| AADC | 48.5 | 108.3 |
| TGM2 | 40 | 149.7 |
| TGM3 | 29 | 297.6 |
| PVALB | 55 | 80.8 |
| TGM4 | 28 | 45.5 |
| CXCL10 | 35 | 66.7 |
| MSRA | 32 | 58.0 |
| Anti-IgG | 10845 | 18165.2 |
| EBNA1 | 206 | 18071.6 |

| **Table S3-** Radiosensitivity test for confirmation of ataxia-telangiectasia in the index patient using a previously published experimental protocol for the G2 assay and G0-micronucleus assay ^2,3^. | | |
| --- | --- | --- |
| **Parameter** | **Studied patient** | **Healthy controls** |
| Chromatid breaks | 54.2* | 14-33 |
| Chromatid gaps | 77.0* | 26-49 |
| Chromosome breaks | 35.7* | 8-28 |
| Chromosome gaps | 23.9 | 9-24 |
| Fragmentations | 14.0* | 5-13 |
| Exchange | 6.3* | 0-4 |
| Micronucleus | 95.4* | 11-24 |

| **Table S4**- Baseline demographic, genetic, and clinical characteristics of the 36 AT patients with SARS-CoV-2 infection in Iranian IEI registry. | | | | | | | |
| --- | --- | --- | --- | --- | --- | --- | --- |
| **ID** | **Age**  **(yr)** | **Sex** | ***ATM* variants** | **Prior noninfectious clinical manifestations** | **Prior infections** | **COVID-19 Severity** |  |
| P1 | 7 | M | Y2371X/ Y2371X | Ataxia, Telangiectasia, Enteropathy, Lymphoproliferation, Autoimmunity, HIgM phenotype | URI, LRI | ICU admission-recovered |  |
| P2 | 10 | F | p.Q513X/Q2689X | Ataxia, Telangiectasia, Enteropathy, Autoimmunity | URI, LRI | Asymptomatic |  |
| P3 | 5 | F | Hom Large del EX62-63 | Ataxia, Enteropathy, Lymphoproliferation, HIgM phenotype | URI | Asymptomatic |  |
| P4 | 12 | M | L1668P/ L1668P | Ataxia, Telangiectasia | URI | Asymptomatic |  |
| P5 | 10 | F | Not tested | Ataxia, Telangiectasia, Enteropathy, | URI | Asymptomatic |  |
| P6 | 7 | M | Q2220X/ Q2220X | Ataxia, Telangiectasia, Lymphoproliferation, Autoimmunity | URI, LRI | Mild-recovered |  |
| P7 | 7 | M | Not tested | Ataxia, Telangiectasia, Enteropathy, Autoimmunity | URI, LRI | Mild-recovered |  |
| P8 | 9 | F | Y1034X/ Y1034X | Ataxia, Telangiectasia, Lymphoproliferation, Autoimmunity | URI, LRI | Mild-recovered |  |
| P9 | 7 | F | Not tested | Ataxia, Telangiectasia, Lymphoproliferation | URI, LRI | Asymptomatic |  |
| P10 | 11 | M | S1905fs*12/ S1905fs*12 | Ataxia, Telangiectasia, Enteropathy, Autoimmunity | URI, LRI | Asymptomatic |  |
| P11 | 7 | M | Not tested | Ataxia, Telangiectasia, Enteropathy, Lymphoproliferation, Autoimmunity | URI, LRI | Asymptomatic |  |
| P12 | 12 | F | Q2220X/ Q2220X | Ataxia, Telangiectasia, Lymphoproliferation | URI, LRI | Asymptomatic |  |
| P13 | 7 | F | E277X/ E277X | Ataxia, Telangiectasia, Enteropathy, Autoimmunity | URI | Asymptomatic |  |
| P14 | 11 | F | L612I/ L612I | Ataxia, Telangiectasia, Lymphoproliferation, Autoimmunity | URI, LRI | Asymptomatic |  |
| P15 | 17 | M | K387Q/ K387Q | Ataxia, Telangiectasia, Enteropathy, Malignancy | URI | Asymptomatic |  |
| P16 | 7 | M | Not tested | Ataxia, Telangiectasia, Enteropathy, Lymphoproliferation | URI, LRI | Asymptomatic |  |
| P17 | 9 | F | L612I/ L612I | Ataxia, Telangiectasia, Lymphoproliferation, Autoimmunity | URI, LRI | Asymptomatic |  |
| P18 | 13 | F | R2151T/ R2151T | Ataxia, Telangiectasia, Lymphoproliferation | URI, LRI | Mild-recovered |  |
| P19 | 7 | M | E2087Kfs*9/ Q2220X | Ataxia, Diarrhea, Autoimmunity, HIgM phenotype | URI, LRI | Mild-recovered |  |
| P20 | 8 | M | c.6198+1G>A/ D2016G | Ataxia, Telangiectasia, Lymphoproliferation, Autoimmunity | URI, LRI | Asymptomatic |  |
| P21 | 5 | F | Q2220X/ Q2220X | Ataxia, Telangiectasia, Enteropathy, Lymphoproliferation | URI, LRI | Asymptomatic |  |
| P22 | 9 | M | c.6807+1G>C/ c.6807+1G>C | Ataxia, Telangiectasia, Enteropathy, Autoimmunity | URI | Asymptomatic |  |
| P23 | 15 | M | c.2921+1G>T/ c.2921+1G>T | Ataxia, Telangiectasia, Enteropathy, Autoimmunity | URI, LRI | Asymptomatic |  |
| P24 | 12 | M | c.2921+1G>T/ c.2921+1G>T | Ataxia, Telangiectasia | URI | Asymptomatic |  |
| P25 | 8 | M | R2151T/ R2151T | Ataxia, Telangiectasia, Enteropathy, Autoimmunity | URI, LRI | Asymptomatic |  |
| P26 | 6 | F | Not tested | Ataxia, Enteropathy, Autoimmunity | URI, LRI | Asymptomatic |  |
| P27 | 10 | F | Q1852Pfs*5/ Q1852Pfs*5 | Ataxia, Telangiectasia, Enteropathy, Autoimmunity, HIgM phenotype | URI, LRI | Mild-recovered |  |
| P28 | 12 | M | D2016G/ D2016G | Ataxia, Telangiectasia, Enteropathy, Lymphoproliferation | URI | Asymptomatic |  |
| P29 | 15 | F | D2016G/ D2016G | Ataxia, Telangiectasia, Enteropathy, Lymphoproliferation | URI | Asymptomatic |  |
| P30 | 9 | F | Q163X/ Q163X | Ataxia, Telangiectasia, Lymphoproliferation, Autoimmunity | URI | Asymptomatic |  |
| P31 | 11 | F | K387Q/ K387Q | Ataxia, Telangiectasia, Lymphoproliferation, Autoimmunity | URI, LRI | Asymptomatic |  |
| P32 | 8 | M | Hom Large del EX37-48 | Ataxia, Telangiectasia | URI, LRI | Asymptomatic |  |
| P33 | 9 | M | Hom Large del EX37-48 | Ataxia, Telangiectasia, Enteropathy, Lymphoproliferation | URI, LRI | Asymptomatic |  |
| P34 | 13 | M | H1082Rfs*14/ H1082Rfs*14 | Ataxia, Telangiectasia, Autoimmunity, HIgM phenotype | URI | Asymptomatic |  |
| P35 | 7 | M | G2023R/E277X | Ataxia, Telangiectasia, Enteropathy Autoimmunity | URI, LRI | Mild-recovered |  |
| P36 | 10 | F | L612I/ L612I | Ataxia, Telangiectasia, Enteropathy | URI, LRI | Mild-recovered |  |
| *URI: upper respiratory infections, LRI: lower respiratory infections, HIgM: hyper IgM immunoglobulin profile, Hom: homozygous, M: male, F: female.* | | | | | | |  |

| **Table S5**- Other variants in known IEI genes in the index patient. | | | | | | | | | | | |
| --- | --- | --- | --- | --- | --- | --- | --- | --- | --- | --- | --- |
| **Chr** | **Position** | **Zyg** | **Ref** | **Alt** | **Impact** | **Gene** | **Exon** | **mRNA change** | **Amino acid change** | **MAF** | **CADD score** |
| chr2 | 163123826 | het | C | A | MODERATE | *IFIH1* | 16/16 | c.2962G>T | p.Val988Leu | 0.0004 | 32 |
| chr3 | 10130168 | het | C | T | MODERATE | *FANCD2* | 35/43 | c.3502C>T | p.Pro1168Ser | - | 23 |
| chr5 | 35873646 | het | A | G | MODERATE | *IL7R* | 5/8 | c.602A>G | p.Tyr201Cys | 0.0003 | 25.6 |
| chr5 | 41161898 | het | C | T | MODERATE | *C6* | 10/18 | c.1355G>A | p.Gly452Glu | 0.004 | 23.6 |
| chr9 | 405025 | het | G | T | MODERATE | *DOCK8* | 14/35 | c.1743G>T | p.Met581Ile | - | 17.51 |
| chr11 | 65487508 | het | C | T | LOW | *RNASEH2C* | 3/3 | c.468+8G>A | - | 0.00002 | 8.937 |
| chr14 | 99641360 | het | C | T | MODERATE | *BCL11B* | 3/3 | c.1600G>A | p.Gly534Ser | - | 14.19 |
| chr14 | 106054107 | het | C | T | MODERATE | *IGHA2* | 2/3 | c.412G>A | p.Gly138Ser | 0.00003 | . |
| chr20 | 62322289 | het | G | A | MODERATE | *RTEL1* | 27/36 | c.2545G>A | p.Gly849Ser | 0.00006 | 2.114 |
| chr21 | 34668546 | het | G | A | MODERATE | *IL10RB* | 7/7 | c.862G>A | p.Glu288Lys | - | 20.4 |
| *Chr: chromosome; Zyg: zygosity; Ref: reference allele; Alt: alteration variant; MAF: minor allele frequency in gnomAD database (*[*https://gnomad.broadinstitute.org/)*](https://gnomad.broadinstitute.org/))*; CADD: Combined Annotation Dependent Depletion score.* | | | | | | | | | | | |

**Figure S1-** Schematic illustration of mutations localization in the *ATM* and *TLR7* genes in the index patient


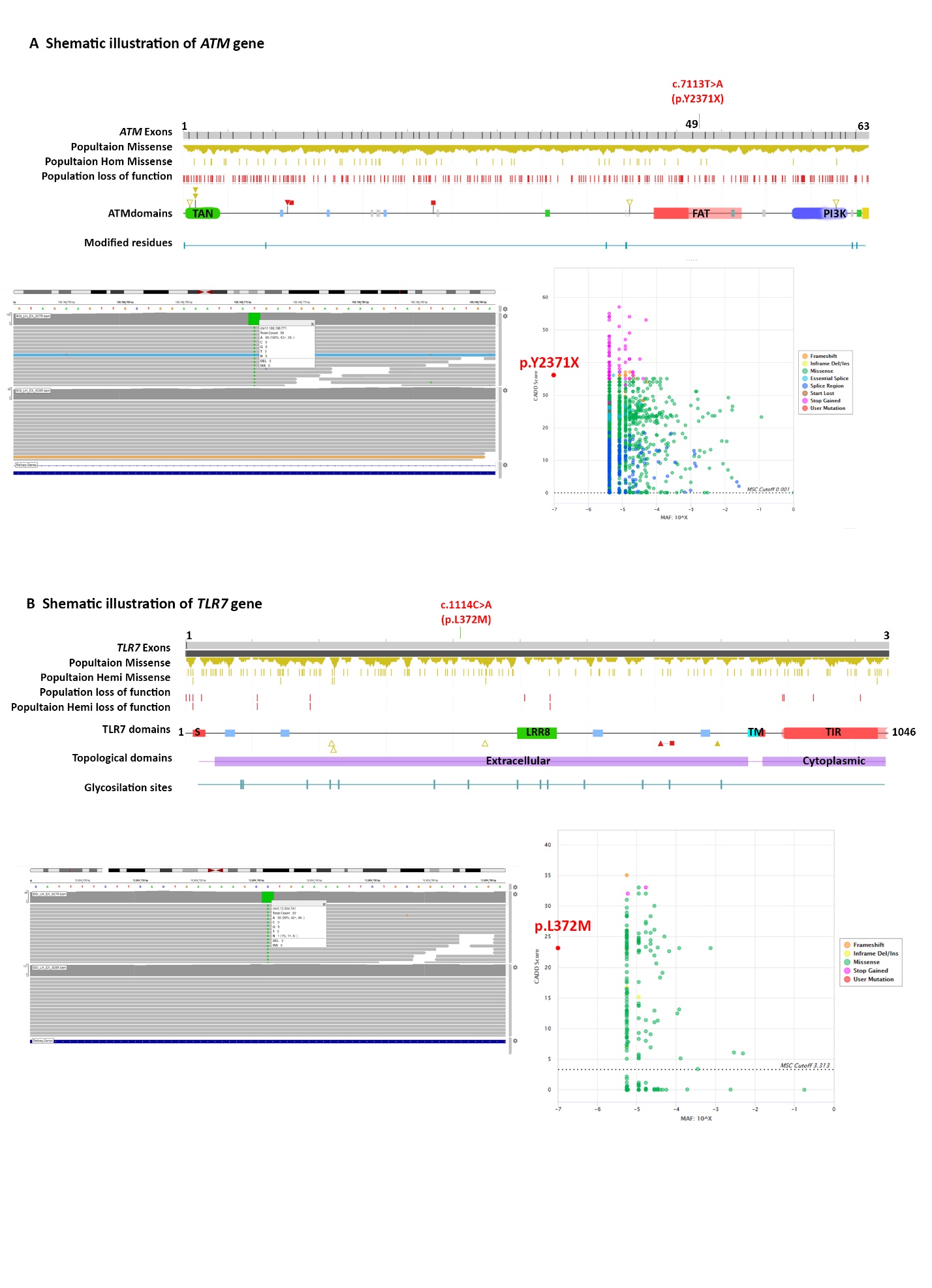


**Supplementary References**

1. Sherina N, Piralla A, Du L, et al. Persistence of SARS-CoV-2-specific B and T cell responses in convalescent COVID-19 patients 6-8 months after the infection. Med (N Y) 2021; 2:281-95.

2. Mahmoodi M, Abolhassani H, Mozdarani H, et al. In vitro chromosomal radiosensitivity in patients with common variable immunodeficiency. Cent Eur J Immunol 2018; 43:155-61.

3. Mozdarani H, Kiaee F, Fekrvand S, et al. G2-lymphocyte chromosomal radiosensitivity in patients with LPS responsive beige-like anchor protein (LRBA) deficiency. Int J Radiat Biol 2019; 95:680-90.
